# Variation in Sagittal Alignment Parameters in Adult Patients before Spine Surgery: A Serial Imaging Study Using Antero-Posterior and Latero-Lateral Projections

**DOI:** 10.3390/diagnostics11112141

**Published:** 2021-11-18

**Authors:** Hunjong Lim, Eugene Lee, Joon-Woo Lee, Bo-Ram Kim, Yusuhn Kang, Joong-Mo Ahn

**Affiliations:** Department of Radiology, Seoul National University Bundang Hospital, 82 Gumi-ro173 Beon-gil, Bundanggu, Seongnam-si 13620, Korea; limhunjong@naver.com (H.L.); joonwoo2@gmail.com (J.-W.L.); boram7072@gmail.com (B.-R.K.); yskang0114@gmail.com (Y.K.); joongmoahn@gmail.com (J.-M.A.)

**Keywords:** EOS, low back pain, radiographic parameters, serial imaging, sagittal spinal alignment

## Abstract

Sagittal parameters of the spine are closely related to the evaluation and treatment of spine disease. However, there has been little research on variations in preoperative sagittal spinal alignment. This study was conducted to assess the variation in sagittal spinal alignment on serial antero-posterior and latero-lateral projections (EOS imaging) in adult patients before spine surgery. The sagittal parameters of 66 patients were collected from two serial images. Comparison between the first and second sagittal parameters was evaluated using the Wilcoxon signed-rank test. Subgroup analysis was performed based on the time interval between radiographs, patient’s age, and type of surgery. The sagittal vertical axis (SVA) exhibited statistically significant changes (*p* = 0.023), with the mean SVA increasing statistically (61.7 mm vs. 73.6 mm) and standard deviation increasing (51.5 mm vs. 61.6 mm) in the second image. Subgroup analysis showed significant differences in SVA (*p* = 0.034) in patients with an interval of >3 months; statistical differences in borderline levels in the SVA (*p* = 0.049) were observed in patients aged >65 years. Other parameters did not show statistically significant differences, except for SVA. Furthermore, SVA differences were statistically significant with increases in the EOS interval (>3 months) and patient age (>65 years).

## 1. Introduction

Regarding spine surgery, evaluating spinal alignment before surgery is routinely performed because there are reports of a significant relationship between sagittal alignment and postoperative patient-reported outcome scores [1,2,3]. The current first choice for assessing sagittal alignment is a whole-spine standing lateral radiograph, which is simple, easy to access, cost-effective, and involve a single brief exposure [4]. In the last few years, antero-posterior and latero-lateral projections (EOS imaging system) have been made available for orthopedic applications. The EOS^®^ slot scanner (EOS imaging, Paris, France) is a proprietary imaging technique based on ultrasensitive X-ray detection technology, which was launched in 2007. This imaging modality depicts the patient’s natural, weight-bearing posture by taking frontal and lateral images of the patient’s body with a very low radiation dose (50–80% less than conventional X-rays) and high image quality [5]. Unlike conventional radiography systems, EOS images do not cause distortions between the center and edges of the radiograph, because linear X-ray sources and detector arrays move together [6]. This full-body EOS enables evaluation of the global alignment of the spine, pelvis, and lower limbs, which is important in diagnosing spinal deformity and surgical outcomes [7,8,9,10,11]. Hence, EOS images are increasingly becoming routine before spine surgery [12].

For preoperative image analysis, reliability and reproducibility should be high. A recent study of whole-body alignment in adolescent idiopathic scoliosis (AIS) reported that most parameters measured by serial EOS images in this group are generally reproducible [13,14]. A study of EOS-based measurement of the sagittal alignment of the spine and pelvis in patients without congenital anomaly, deformity, or previous history of spine and pelvis surgery showed excellent intra-rater and inter-rater reliability [15]. In adult patients with lower extremity mechanical axis malalignment, EOS-based measurements were reproducible and showed excellent statistical reliability, comparable to the gold standard of conventional radiographs [16].

However, another study comparing serial whole-body sagittal alignment images in adult patients with mild low back pain showed that the sagittal vertical axis (SVA) has the largest variation between individuals of low pelvic tilt (PT) due to reflections of dynamism in spinal balancing [17]. There are limited data on the interval difference of sagittal parameters for preoperative assessments in adult patients with severe pain requiring surgery. If the reproducibility of images performed serially for the preoperative analysis is low, this will cast doubt on the reliability of EOS-based measurements for accurate surgical planning.

There is a need to analyze the possible changes in measurement parameters when EOS is taken at regular intervals in patients with sufficient pain who undergo surgery. Moreover, if statistically significant interval changes between two measurements can occur over a certain period, it may be necessary to retake the EOS images shortly before surgery. Therefore, this study assessed the variations in sagittal spinal alignment on serial EOS imaging in adult patients before spine surgery.

## 2. Materials and Methods

### 2.1. Study Population

We retrospectively searched the electronic medical record system and Radiology Department database at our institution between November 2017 and May 2020 for cases meeting the following inclusion criteria: (1) adult patients who underwent spinal surgery due to degenerative spinal diseases or deformity correction; (2) uncontrolled severe pain or radiculopathy despite sufficient conservative treatments; and (3) availability of serial pre-operative EOS imaging. We excluded patients with: (1) tumor, trauma, congenital anomaly, or infection; (2) prior spinal surgery; (3) coexisting spinal cord disorders which can cause motor dysfunction; and (4) inappropriate EOS images due to inconsistent posture, not-completely covered whole spine, severe artifacts, and the usage of support devices. Finally, 66 patients (18 men, 48 women; mean age, 68.3 (range 53–83) years) were enrolled in this study.

Using the EOS^®^ system, consisting of two co-linked pairs of a 45 cm wide linear radiation source and detector, simultaneous anteroposterior (AP) and lateral images were recorded. The sources were coupled to linear detectors using the micromesh gaseous structure technology [15]. These spatially calibrated biplanar images enable precise three-dimensional reconstruction of the skeletal system by avoiding conical projection. Additionally, a Xenon multiwire proportional chamber was placed at 1.3 m from the X-ray source, with the patient standing approximately 1 m from the source. When a low-dose X-ray beam passed through the patients and the chamber, each X-ray beams generated a secondarily increased photon flow within the chamber by the localized cascade of ionization, known as a Townsend discharge. In this principle, EOS can cover a field of view of 180 × 45 cm in a single acquisition with a low dose of primary X-ray beam, resulting in high-quality, high-contrast X-ray images [6].

### 2.2. EOS Examination

Patients were instructed to hold their breath while maintaining a fixed weight-bearing standing position (stand up straight, look horizontally, touch maxillary sinus with fingers, not lean against the wall or hold the wall). The user determined the start- and endpoints of the vertical scan (up to 175 cm) to minimize radiation exposure to body parts. Two perpendicular X-ray beams and corresponding detectors traveled vertically in 8 to 15 s, depending on the patient’s height. Trained radiographers with >1 year of experience recorded the images. All radiographs were uploaded to the hospital’s server and digitalized into PACS (Picture Archiving and Communication System).

### 2.3. EOS Measurement

**Figure 1:**
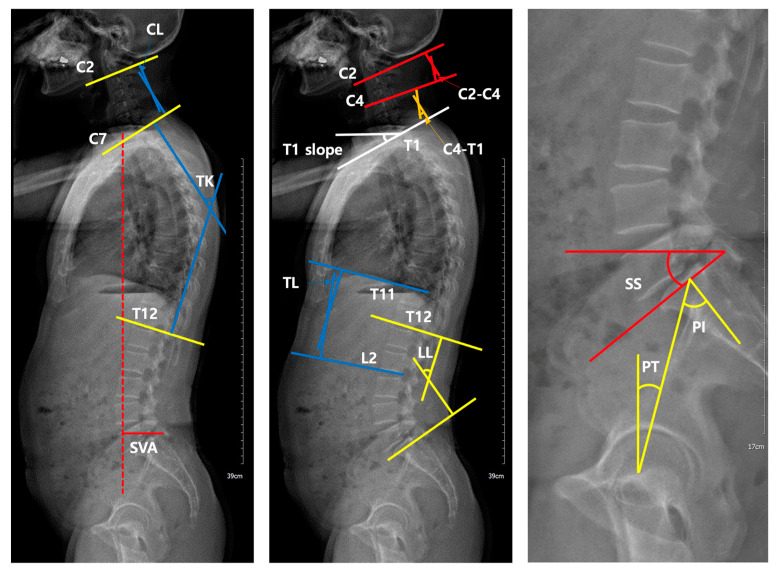
Measurements of sagittal parameters using the EOS imaging system. SVA (sagittal vertical axis), CL (cervical lordosis), TK (thoracic kyphosis), TL (thoracolumbar angle), LL (lumbar lordosis), SS (sacral slope), PT (pelvic tilt), PI (pelvic incidence), T1 slope (angle between a horizontal line and the superior endplate of T1), C2–C4 (Cobb’s angle between the inferior endplates of C2 and C4), and C4–T1 (Cobb’s angle between the inferior endplates of C2 and C4).

Using lateral EOS images, we measured sagittal radiographic parameters, including the sagittal vertical axis (SVA), cervical lordosis (CL), thoracic kyphosis (TK), thoracolumbar angle (TL), lumbar lordosis (LL), sacral slope (SS), pelvic tilt (PT), pelvic incidence (PI), T1 slope, C2–C4, and C4–T1 (Figure 1) [18]. SVA was measured as the length of a horizontal line connecting the posterosuperior corner of S1 to the vertical plumbline from the center of the C7 vertebral body. CL is Cobb’s angle between the inferior endplates of C2 and C7. TK is Cobb’s angle between the inferior endplates of C7 and T12. TL is Cobb’s angle between the superior endplate of T11 and the inferior endplate of L2. LL is Cobb’s angle between the inferior endplate of T12 and the superior endplate of S1. SS is the angle between the superior plate of S1 and a horizontal line. PT is the angle between the vertical reference line and the line connecting the bicoxofemoral axis midpoint to the sacral plate midpoint. PI was measured as an angle between the line connecting the midpoint of the bicoxofemoral axis to the midpoint of the sacral plate and the line perpendicular to the sacral plate. T1 slope is the angle between a horizontal line and the superior endplate of T1. C2–C4 is Cobb’s angle between the inferior endplates of C2 and C4. C4–T1 is Cobb’s angle between the inferior endplates of C4 and T1.

All measurements were performed independently by two radiologists (a second-year radiology resident and musculoskeletal radiologist with nine years of experience), and an average of their readings was recorded. We directly measured the obtained images using a software tool provided by PACS. Additionally, all measurements were recorded with significant figures to the first decimal place.

### 2.4. Statistical Analysis

Statistical analyses were performed using the Statistical Package for the Social Sciences (SPSS, Chicago, IL, USA), version 23 software. The first and second sagittal radiographic parameters were compared using the Wilcoxon signed-rank test. Similar tests were performed after the stratification of patients based on the time interval between radiographs (interval imaging < 3 months vs. >3 months), patient’s age (age ≤ 65 vs. age > 65), and type of surgery (deformity correction or not). A *p*-value of <0.05 was considered statistically significant.

## 3. Results

### 3.1. Patients Demographics

In total, 132 whole-body sagittal images of 66 patients were analyzed. The preoperative average visual analogue scale (VAS) score was 7.1. Long-level deformity correction surgery was performed in 18 patients with spinal curvature/alignment disorder, whereas others underwent decompression with or without fusion depending on the lesion, at one level or multiple levels. The mean interval between the first and second EOS images was 112 days [standard deviation (SD) 116], and the time interval between the first/second EOS data point and surgery was 139.6 ± 27.6 days.

The mean standard spinopelvic parameters—PI, PT, and SS—in this group were 51.6° (SD 12.3), 26.7° (SD 12.8), and 25.1° (13.1), respectively. Moreover, the mean values of SVA and T1 slopes were 67.7 mm (SD 56.9) and 20.1° (SD 7.7), respectively (Table 1).

### 3.2. Changes in Sagittal Alignment Parameters

**Figure 2:**
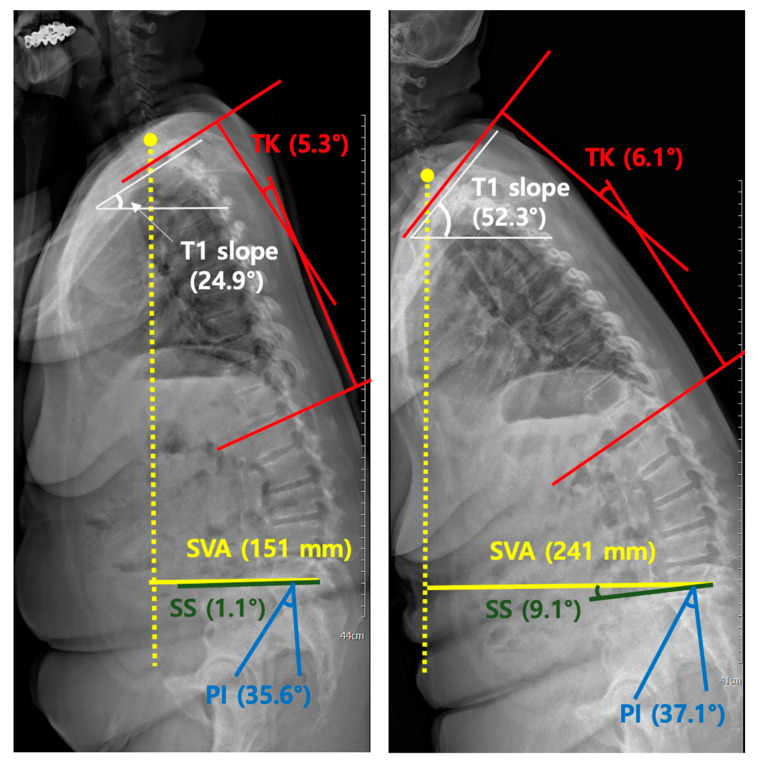
Changes in the sagittal vertical axis (SVA, length of a horizontal line connecting the posterosuperior corner of S1 to vertical plumbline from center of the C7 vertebral body) and T1 slope (angle between a horizontal line and the superior endplate of T1) on serial EOS images. Radiographs of a representative patient showing significant changes in the sagittal vertical axis (SVA, yellow) and T1 slope (white). There was no significant change in thoracic kyphosis (TK, Cobb’s angle between the inferior endplates of C7 and T12, red), sacral slope (SS, angle between the superior plate of S1 and a horizontal line, green), and pelvic incidence (PI, angle between the line connecting the midpoint of the bicoxofemoral axis to the midpoint of the sacral plate and the line perpendicular to the sacral plate, blue) between the two EOS images (time interval between the first and second EOS images is 140 days, the patient underwent surgery 33 days after taking the second EOS).

As shown in Table 2, of the sagittal alignment parameters, only SVA showed a statistically significant change (*p* = 0.023), with the mean SVA increasing statistically (61.7 vs. 73.6) and SD increasing (51.5 vs. 61.6) in the second EOS image before surgery (Figure 2).

### 3.3. Subgroup Analysis of Sagittal Alignment Parameters

Time-interval-based (three months) subgroup analysis (Table 3) showed a significant difference in SVA in patients with EOS images recorded at an interval of >3 months (*p* = 0.034). However, SVA showed no statistically significant difference (*p* = 0.357) when the time interval of EOS was <three months. The T1 slope showed a statistical difference in borderline levels (*p* = 0.048) in patients with <3 months of the interval, but not in those with >3 months.

Patient’s age-based (65 years) subgroup analysis (Table 4) showed only borderline differences in SVA in patients aged >65 years (*p* = 0.049). Other parameters showed no significant differences in age.

According to the surgical method based on deformity corrections, none of the parameters showed any significant differences (Table 5).

Patients in the non-deformity correction group were divided into one level (*n* = 26; fusion (*n* = 22) and depression only (*n* = 4)) and more than two levels (*n* = 22; all fusion) according to the surgical level. This showed only borderline differences in C4–T1 in patients who underwent multilevel fusion surgery (*p* = 0.042). In other parameters, there were no statistically significant differences between the two groups. All patients in the deformity correction group received a long-level fusion from the lower thoracic spine to the iliac bone for major curve correction and did not perform additional subgroup analysis (Table 6).

## 4. Discussion

In the evaluation and treatment of spinal disease, the sagittal balance of the spine and pelvis is the most important [19]. Conventionally, for sagittal curvature evaluation, whole-spine standing lateral radiography is widely performed based on its easy usability and simplicity, showing good intra-observer reliability and a good-to-excellent inter-rater reproducibility [20]. However, whole-spine standing lateral radiography has several limitations. The distortion between the center and edges of the radiograph results in measurement errors due to the enlargement of structures located far from the central region [21]. To assess the whole lateral view, it is necessary to combine split images, with the possibility of distortion. The patient’s movements during radiography induced by relatively long sagittal image acquisition times cause a change in sagittal alignment. After developing EOS, these limitations were overcome by allowing a single-shot image and a shorter acquisition time (8 to 15 s), presenting the possibility of low-dose radiation exposure. According to recently published clinical reports, the EOS average skin dose was reduced from six to nine times in the thoracoabdominal region compared with that of computed radiography [21]. The dose-area product of EOS is as low as 38% of that of standard digital radiography systems [22]. With the aid of paired X-ray sources and detectors that move in tandem, EOS has no geometric magnification. Moreover, EOS does not need to stitch multiple images together to produce complete spinal lateral images due to its ability to scan a full-body image. Regarding Cobb’s angle measurements, EOS provides comparable accuracy and reproducibility in both pre- and post-operative adolescent idiopathic scoliosis patients compared with CT [23,24]. Furthermore, EOS gives lower labor costs per examination due to the shorter examination time (248 s to complete EOS vs. 449 s for standard digital radiography) and greater patient comfort regarding noise, compared with standard digital radiographs [22]. For these reasons, EOS imaging is an attractive alternative to conventional radiography for spine or whole-body alignment evaluation [25].

However, before using this new low-radiation-dose X-ray device, the accuracy and reliability should be checked. In a study by Kim et al., the EOS system showed excellent intra-rater (intraclass correlation coefficients (ICCs) ranging from 0.898 to 0.982) and interrater reliability (ICC ranging from 0.794 to 0.837) in the assessment of the sagittal alignment of the spine and pelvis [15]. A recent study measuring all sagittal radiographic parameters in patients with first-episode mild low back pain to describe normal variation showed small mean variations (<1°), except for in pelvic tilt (PT) (1.2°), C2–T1 alignment (1.2°), and SVA (2.9 cm) [17]. This result guarantees the reliability of EOS imaging in normal adult patients and raises the possibility of inconsistency in some parameters due to the dynamic nature of body balance [26]. However, no previous studies have been conducted on patients who require surgical treatment, and clinically, EOS is often part of a preoperative analysis, which necessitates research on patients who underwent surgery. In our study, most sagittal alignment parameters on serial EOS imaging did not show statistically significant interval changes even in patients requiring surgical treatment, which can be interpreted in the same context as the results of a previous study [17].

The difference in existing results in our work is that SVA showed statistically significant interval variations (*p* = 0.023). This variation in SVA was shown in subgroup analyses when the EOS retake interval was >3 months and based on age; borderline significance was shown at an advanced age of 65 years. Interestingly, variations were not apparent in patients with short time intervals or relatively young patients with serial EOS images. In the first group (≤65 years), the mean value of SVA in the second radiograph showed a difference from that of the first (66.6 vs. 96.4), which is thought to have occurred due to large differences in SVA in 3 out of 15 patients. The SVA differences in the three patients were 239.0, 111.9, and 89.9 mm, respectively. In the rest of the patients in group 1, the changes in SVA between the first and second radiographs were not significant, which resulted in no statistically significant differences overall.

SVA has several clinically important significances and must be strictly evaluated before surgery. Generally, sagittal spine alignment is affected by age. There are two points of importance: increasing “positive spinal balance” due to a loss of lumbar lordosis with a more forward sagittal vertical axis [27] and increasing thoracic kyphosis with advancing age [28]. In a retrospective study correlating radiographic measurements with clinical symptoms in adult scoliosis, a positive sagittal balance was the most important and significant predictor of clinical symptoms, regardless of previous surgery [29]. Therefore, the surgeon should plan for a more normal sagittal balance of the spine as an important goal of reconstructive spine surgery. According to a recent study assessing the effect of sagittal spino-pelvic alignment on the clinical symptoms of thoracic, lumbar kyphosis (TLK) in osteoporotic patients, sagittal imbalance (SVA > 5 cm) was more closely related to clinical symptoms than other radiological parameters [30]. The main purpose of surgery for kyphosis is reducing deformity, pain, and neurological symptoms, and preventing the curve from worsening. Therefore, the sagittal imbalance should be considered in the management of TLK in osteoporotic patients. SVA is the most important parameter not only before surgery, but also after surgery, influencing the clinical outcome of patients surgically treated for degenerative lumbar spondylolisthesis [31]. Overall postoperative sagittal balance, as defined by SVA, determines the postoperative patient-rated outcome.

The variations in SVA can be attributed to multiple factors, the first of which is that SVA itself has a large interval variation. A previous study [17] reporting the normal variation in sagittal spinal alignment parameters concluded that SVA has the largest variation in individuals with low pelvic tilt, suggesting a wide range of normality. In our patients, SVA exhibited a wide range and large SDs, which is consistent with these results. In our study, the mean SVA was measured at 67.7 mm, which is significantly higher than that in the normal population. This may be attributed to the difficulty in sagittal balancing due to stooping postures or severe pain in patients requiring surgical treatment, which may have caused statistical differences. According to a study by Steven et al. [32], in patients with spinal deformity, positive SVA correlates with the severity of symptoms in a linear fashion.

Furthermore, compared to a similar study performed by Hey et al. [4] in patients with adolescent idiopathic scoliosis (AIS) and a previously mentioned report [17], our study showed predictable differences in SVA between the first and second EOS recorded serially. The analysis was based on three months’ records because the patient’s regular follow-up period in our institution is usually three months, and the average serial EOS interval in this study is approximately over three months. These results can also be attributed to the wide variations in the SVA or to the increasing degree of pain as the timing of surgery approaches. The borderline significance seen in older patients can be interpreted as reflecting complex and various sagittal balancing in advanced ages. Consequently, these results suggest that the interpretation of SVA results in patients who are about to undergo surgery should be considered; the measurement values may show statistically significant differences, which requires attention to conclude with a single image.

Excluding the differences related to SVA, the T1 slope in patients with X-rays recorded <3 months apart (*p* = 0.048) and the C4–T1 in patients underwent multilevel fusion surgery remained the only borderline significant difference. We believe that the difference could be due to the following reason: radiographically, some parameters related to C7 and T1 are difficult to measure because there are overlapping points. Due to overlapping with surrounding chest wall structures, the T1 slope was the most difficult parameter to measure [15]. In cervical spondylotic myelopathy, cervical sagittal imbalance, as defined by a C2–C7 SVA (the distance from a plumb line drawn at the midpoint at the base of C2 to that drawn at the midpoint of the base of C7) of >40 mm, is associated with worse postoperative clinical outcomes after surgery, resulting in <50% of patients clinically improving after cervical spine surgery [33]. According to a study performed by Chong et al., T1 slope is the only parameter that demonstrated a significant correlation with both Cobb’s angles of C2–C7 and C2–C7 SVA in symptomatic patients [34]. In our study, T1 slope and C4–T1 showed borderline differences in variation, but in patients subject to cervical surgery where cervical sagittal balance is essential, it may be necessary to attend to the interpretation of T1 slope measurements, similar to SVA. However, because the subjects of this study were patients who underwent surgery due to symptoms in the thoracolumbar region, additional consideration of the variation of the T1 slope or the lower cervical alignment C4–T1 will be needed in patients who undergo surgery due to degenerative cervical diseases.

The limitations of this study can be grouped into four aspects. First, measurement errors were caused. EOS is photographed with low-dose radiation; therefore, the resolution may be poor, and measurement errors may have occurred depending on the investigator. Secondly, although the patients were requested to assume a proper position during X-ray exposure, it might have been difficult for elderly patients. As such, only patients with no significant posture difference between two serial images were involved in this study. We excluded 14 inappropriate EOS images out of 80 images due to inconsistent posture, causing a not-completely covered whole spine (*n* = 9), severe artifacts (*n* = 3), and usage of support devices (*n* = 2). Especially, in the case of inconsistent posture, most of the patients who underwent deformity correction operations presented with severe stooping. As a result, at least one of the two EOS images did not cover a portion of the cervical spine. This may raise the possibility of a selection bias. Thirdly, selection bias could have occurred because, unlike plain radiography performed as a routine process, the EOS was conducted in selective patients. In our country, where the study was conducted, EOS imaging is not yet covered by medical insurance. Therefore, it is not implemented routinely, and EOS does not reflect the entire cohort. The final limitation of the study pertains to the relatively small sample size. Although there were no statistical errors, this possibly resulted in no significant differences between the first and second images. The small number of patients also entails limitations because they were not classified according to the etiology of the pain. Subsequently, an increase in the number of patients and subgroup analysis is required according to each etiology.

## 5. Conclusions

Most radiographic sagittal spinal parameters in patients who are about to undergo surgery are generally reproducible. Our study showed that SVA was varied in the serial EOS images, and this difference was statistically significant as the EOS interval increased (>3 months) and as the patients aged (>65 years). Therefore, in these patient populations, attention is needed, especially in the interpretation of parameter measurements of SVA. In the patient group where SVA is important surgically, it is necessary to consider repeating the preoperative imaging study.

## Figures and Tables

**Table 1:** Patient characteristics in terms of radiographic parameters of the spine of all EOS images.

Parameter ^1^	All Patients (*n* = 66) and Radiographic Parameters (*n* = 132 Images)		
	Mean	Range	Standard Deviation
SVA (mm)	67.7	−43.8–265.3	56.9
CL (°)	11.9	0.1–54.0	11.3
TK (°)	23.8	0.2–53.5	14.1
TL (°)	13.4	0.1–63.6	11.8
LL (°)	31.7	0.2–92.4	19.8
SS (°)	25.1	1.1–80.6	13.1
PT (°)	26.7	0.2–62.6	12.8
PI (°)	51.6	23.5–94.0	12.3
T1 slope (°)	20.1	1.2–52.3	7.7
C2–C4 (°)	8.2	0.0–31.4	6.7
C4–T1 (°)	8.8	0.1–31.3	7.2

^1^ SVA (sagittal vertical axis), CL (cervical lordosis), TK (thoracic kyphosis), TL (thoracolumbar angle), LL (lumbar lordosis), SS (sacral slope), PT (pelvic tilt), PI (pelvic incidence), T1 slope (angle between a horizontal line and the superior endplate of T1), C2–C4 (Cobb’s angle between the inferior endplates of C2 and C4), and C4–T1 (Cobb’s angle between the inferior endplates of C2 and C4).

**Table 2:** Difference in radiographic measurements in the first and second EOS images.

Parameter ^1^	All Patients (*n* = 66)						
	First EOS			Second EOS			*p*
	Mean	Range	SD ^3^	Mean	Range	SD ^3^	
SVA (mm)	61.7	−23.9–221.9	51.5	73.6	−43.8–265.3	61.6	0.023 ^2^
CL (°)	11.7	0.1–52.1	11.4	12.1	0.2–54.0	11.4	0.846
TK (°)	23.8	0.2–53.5	13.5	23.8	0.2–51.8	14.8	0.662
TL (°)	13.4	0.4–63.6	12.1	13.5	0.1–63.3	11.6	0.982
LL (°)	31.9	2.9–92.4	20.1	31.4	0.2–79.3	19.6	0.387
SS (°)	25.2	1.1–80.6	13.4	25.1	2.0–69.6	12.8	0.671
PT (°)	26.7	3.3–62.6	13.5	26.8	0.2–61.6	12.3	0.881
PI (°)	51.6	23.5–94.0	12.3	51.6	32.4–87.6	12.5	0.921
T1 slope (°)	20.4	3.2–37.4	7.2	19.7	1.2–52.3	8.3	0.399
C2–C4 (°)	8.3	0.0–31.4	7.3	8.0	0.6–27.3	6.0	0.609
C4–T1 (°)	8.8	0.1–31.3	7.4	8.8	0.1–28.9	7.1	0.461

^1^ SVA (sagittal vertical axis), CL (cervical lordosis), TK (thoracic kyphosis), TL (thoracolumbar angle), LL (lumbar lordosis), SS (sacral slope), PT (pelvic tilt), PI (pelvic incidence), T1 slope (angle between a horizontal line and the superior endplate of T1), C2–C4 (Cobb’s angle between the inferior endplates of C2 and C4), and C4–T1 (Cobb’s angle between the inferior endplates of C2 and C4). ^2^ Statistically significant difference. ^3^ Standard deviation.

**Table 3:** Subgroup analysis based on the time interval between EOS <3 and >3 months apart.

Parameter ^1^	Group 1 (<3 Months) Patients (*n* = 38)					Group 2 (>3 Months) Patients (*n* = 28)				
	First X-ray (*n* = 38)		Second X-ray (*n* = 38)		*p*	First X-ray (*n* = 28)		Second X-ray (*n* = 28)		*p*
	Mean	SD ^3^	Mean	SD ^3^		Mean	SD ^3^	Mean	SD ^3^	
SVA (mm)	61.3	51.9	65.9	55.9	0.357	62.3	51.8	84.0	68.3	0.034 ^2^
CL (°)	10.8	11.8	12.1	12.8	0.936	12.8	10.8	12.0	9.4	0.716
TK (°)	24.0	12.8	23.0	14.6	0.321	23.4	14.6	24.9	15.4	0.665
TL (°)	12.8	11.1	12.9	9.5	0.988	14.3	13.4	14.3	14.1	0.946
LL (°)	32.1	21.1	30.5	20.3	0.153	31.7	18.9	32.8	19.0	0.802
SS (°)	25.5	14.0	25.3	12.3	0.885	24.7	12.8	24.8	13.7	0.524
PT (°)	24.9	11.7	24.5	10.5	0.722	29.1	15.5	29.9	13.9	0.624
PI (°)	50.6	11.1	49.6	10.1	0.103	52.8	13.8	54.4	14.9	0.172
T1 slope (°)	21.8	7.0	19.4	7.2	0.048 ^2^	18.5	7.1	20.2	9.7	0.387
C2–C4 (°)	8.6	7.5	8.2	6.6	0.766	8.0	7.2	7.8	5.3	0.690
C4–T1 (°)	8.1	7.0	7.1	6.1	0.868	9.6	7.9	11.2	7.7	0.236

^1^ SVA (sagittal vertical axis), CL (cervical lordosis), TK (thoracic kyphosis), TL (thoracolumbar angle), LL (lumbar lordosis), SS (sacral slope), PT (pelvic tilt), PI (pelvic incidence), T1 slope (angle between a horizontal line and the superior endplate of T1), C2–C4 (Cobb’s angle between the inferior endplates of C2 and C4), and C4–T1 (Cobb’s angle between the inferior endplates of C2 and C4). ^2^ Statistically significant difference. ^3^ Standard deviation.

**Table 4:** Subgroup analysis based on the patient’s age between age ≤65 and >65 years apart.

Parameter ^1^	Group 1 (≤65 Years) Patients (*n* = 15)					Group 2 (>65 Years) Patients (*n* = 51)				
	First X-ray (*n* = 15)		Second X-ray (*n* = 15)		*p*	First X-ray (*n* = 51)		Second X-ray (*n* = 51)		*p*
	Mean	SD ^3^	Mean	SD ^3^		Mean	SD ^3^	Mean	SD ^3^	
SVA (mm)	66.6	56.4	96.4	96.4	0.233	60.3	50.5	66.9	46.2	0.049 ^2^
CL (°)	15.0	13.2	12.4	9.7	0.394	10.7	10.7	12.0	11.9	0.866
TK (°)	28.9	15.7	28.9	17.1	0.496	22.3	12.5	22.3	13.9	0.779
TL (°)	8.3	7.5	9.4	7.4	0.334	14.9	12.8	14.7	12.4	0.530
LL (°)	33.5	24.1	37.3	22.3	0.156	31.5	19.0	29.7	18.6	0.072
SS (°)	23.7	15.2	22.9	14.3	0.427	25.7	13.0	25.7	12.5	1.000
PT (°)	28.9	17.5	30.4	13.6	0.307	26.0	12.2	25.7	11.8	0.718
PI (°)	53.8	11.8	53.5	12.0	0.650	50.9	12.5	51.1	12.7	0.933
T1 slope (°)	20.5	8.1	23.2	11.1	0.100	20.3	7.0	18.7	7.1	0.100
C2–C4 (°)	9.0	7.9	8.9	7.2	0.955	8.1	7.2	7.8	5.7	0.558
C4–T1 (°)	8.8	8.1	10.8	7.7	0.112	8.8	7.2	8.3	6.8	0.970

^1^ SVA (sagittal vertical axis), CL (cervical lordosis), TK (thoracic kyphosis), TL (thoracolumbar angle), LL (lumbar lordosis), SS (sacral slope), PT (pelvic tilt), PI (pelvic incidence), T1 slope (angle between a horizontal line and the superior endplate of T1), C2–C4 (Cobb’s angle between the inferior endplates of C2 and C4), and C4–T1 (Cobb’s angle between the inferior endplates of C2 and C4). ^2^ Statistically borderline differences. ^3^ Standard deviation.

**Table 5:** Subgroup analysis based on surgical methods.

Parameter ^1^	Group 1 (Non-Deformity Correction) Patients (*n* = 48)					Group 2 (Deformity Correction) Patients (*n* = 18)				
	First X-ray (*n* = 48)		Second X-ray (*n* = 48)		*p*	First X-ray (*n* = 18)		Second X-ray (*n* = 18)		*p*
	Mean	SD ^2^	Mean	SD ^2^		Mean	SD ^2^	Mean	SD ^2^	
SVA (mm)	47.0	40.6	54.7	44.4	0.071	101.0	57.6	123.9	73.5	0.170
CL (°)	9.3	7.7	10.1	9.4	0.870	18.1	16.5	17.3	14.4	0.811
TK (°)	26.1	13.6	25.5	14.6	0.492	17.6	11.4	19.5	15.0	0.811
TL (°)	12.6	12.7	12.8	12.5	0.947	15.5	10.3	15.4	8.7	0.948
LL (°)	36.7	18.0	35.7	18.3	0.291	19.2	20.1	20.1	19.0	0.913
SS (°)	28.1	10.5	28.3	10.6	0.988	17.5	17.2	16.4	14.4	0.500
PT (°)	22.6	10.1	22.5	9.6	0.637	37.6	15.4	38.1	11.6	0.777
PI (°)	50.4	11.0	51.0	12.4	0.486	54.8	15.0	53.3	13.0	0.199
T1 slope (°)	19.5	6.5	19.2	7.1	0.874	22.6	8.6	21.3	10.8	0.327
C2–C4 (°)	7.4	5.7	6.9	4.9	0.470	10.8	10.3	11.1	7.8	0.811
C4–T1 (°)	7.2	6.2	8.0	6.7	0.145	13.3	8.6	11.1	7.7	0.446

^1^ SVA (sagittal vertical axis), CL (cervical lordosis), TK (thoracic kyphosis), TL (thoracolumbar angle), LL (lumbar lordosis), SS (sacral slope), PT (pelvic tilt), PI (pelvic incidence), T1 slope (angle between a horizontal line and the superior endplate of T1), C2–C4 (Cobb’s angle between the inferior endplates of C2 and C4), and C4–T1 (Cobb’s angle between the inferior endplates of C2 and C4). ^2^ Standard deviation.

**Table 6:** Subgroup analysis based on one level versus multiple levels within Group 1 (non-deformity correction) patients.

Parameter ^1^	Group 1 (One Level) Patients (*n* = 26)					Group 2 (Multilevel) Patients (*n* = 22)				
	First X-ray (*n* = 26)		Second X-ray (*n* = 26)		*p*	First X-ray (*n* = 22)		Second X-ray (*n* = 22)		*p*
	Mean	SD ^3^	Mean	SD ^3^		Mean	SD ^3^	Mean	SD ^3^	
SVA (mm)	37.8	42.0	47.0	45.0	0.238	57.8	37.0	64.0	42.7	0.178
CL (°)	9.6	8.4	10.5	9.9	0.889	8.9	7.0	9.5	9.0	0.884
TK (°)	27.6	12.7	27.0	14.1	0.381	24.3	14.6	23.6	15.2	0.884
TL (°)	12.8	14.5	12.8	15.0	0.829	12.4	10.3	12.7	9.2	0.833
LL (°)	40.5	18.3	38.7	18.4	0.131	32.3	17.0	32.2	17.9	0.935
SS (°)	31.6	9.7	31.4	9.8	0.657	23.9	9.9	24.8	10.6	0.661
PT (°)	20.1	8.8	20.5	9.2	0.509	25.4	10.9	25.0	9.7	0.884
PI (°)	50.9	13.0	51.9	13.6	0.395	49.8	8.4	49.9	10.9	0.910
T1 slope (°)	19.4	5.9	20.4	5.9	0.248	19.6	7.3	17.7	8.3	0.223
C2–C4 (°)	6.8	6.0	6.1	4.5	0.648	8.1	5.5	7.8	5.2	0.592
C4–T1 (°)	7.6	6.0	8.2	7.4	0.713	6.6	6.5	7.7	5.9	0.042 ^2^

^1^ SVA (sagittal vertical axis), CL (cervical lordosis), TK (thoracic kyphosis), TL (thoracolumbar angle), LL (lumbar lordosis), SS (sacral slope), PT (pelvic tilt), PI (pelvic incidence), T1 slope (angle between a horizontal line and the superior endplate of T1), C2–C4 (Cobb’s angle between the inferior endplates of C2 and C4), and C4–T1 (Cobb’s angle between the inferior endplates of C2 and C4). ^2^ Statistically borderline differences. ^3^ Standard deviation.

## Data Availability

The data presented in this study are available on request from the corresponding author. The data are not publicly available due to privacy.

## Abbreviations

SVA	sagittal vertical axis
CL	cervical lordosis
TK	thoracic kyphosis
TL	thoracolumbar angle
LL	lumbar lordosis
SS	sacral slope
PT	pelvic tilt
PI	pelvic incidence
T1 slope	angle between a horizontal line and the superior endplate of T1
C2–C4	Cobb’s angle between the inferior endplates of C2 and C4
C4–T1	Cobb’s angle between the inferior endplates of C4 and T1
